# Uneven Large-Scale Movement Patterns in Wild and Reintroduced Pre-Adult Bearded Vultures: Conservation Implications

**DOI:** 10.1371/journal.pone.0065857

**Published:** 2013-06-11

**Authors:** Antoni Margalida, Martina Carrete, Daniel Hegglin, David Serrano, Rafael Arenas, José A. Donázar

**Affiliations:** 1 Division of Conservation Biology, Institute of Ecology and Evolution, University of Bern, Bern, Switzerland; 2 Bearded Vulture Study and Protection Group, El Pont de Suert, Lleida, Spain; 3 Department of Physical, Chemical and Natural Systems, Universidad Pablo de Olavide, Sevilla, Spain; 4 Stiftung Pro Bartgeier, Zürich, Switzerland; 5 SWILD - Urban Ecology and Wildlife Research, Zürich, Switzerland; 6 Vulture Conservation Foundation, Zürich, Switzerland, fSWILD-Urban Ecology and Wildlife Research, Zürich, Switzerland; 7 Department of Conservation Biology, Estación Biológica de Doñana, CSIC Sevilla, Spain; 8 Gestión del Medio Natural, Dirección Provincial de Córdoba, Consejería de Medio Ambiente, Córdoba, Spain; The Ohio State University, United States of America

## Abstract

After the quasi-extinction of much of the European vertebrate megafauna during the last few centuries, many reintroduction projects seek to restore decimated populations. However, the future of numerous species depends on the management scenarios of metapopulations where the flow of individuals can be critical to ensure their viability. This is the case of the bearded vulture *Gypaetus barbatus*, an Old World, large body-sized and long-lived scavenger living in mountain ranges. Although persecution in Western Europe restrained it to the Pyrenees, the species is nowadays present in other mountains thanks to reintroduction projects. We examined the movement patterns of pre-adult non-breeding individuals born in the wild population of the Pyrenees (*n* = 9) and in the reintroduced populations of the Alps (*n* = 24) and Andalusia (*n* = 13). Most birds were equipped with GPS-GSM radio transmitters, which allowed accurate determination of individual dispersal patterns. Two estimators were considered: i) step length (i.e., the distance travelled per day by each individual, calculated considering only successive days); and ii) total dispersal distance (i.e., the distance travelled between each mean daily location and the point of release). Both dispersal estimators showed a positive relationship with age but were also highly dependent on the source population, birds in Andalusia and Alps moving farther than in Pyrenees. Future research should confirm if differences in dispersal distances are the rule, in which case the dynamics of future populations would be strongly influenced. In summary, our findings highlight that inter-population differences can affect the flow of individuals among patches (a key aspect to ensure the viability of the European metapopulation of the endangered bearded vulture), and thus should be taken into account when planning reintroduction programs. This result also raises questions about whether similar scenarios may occur in other restoration projects of European megafauna.

## Introduction

European megafauna populations of large body-sized vertebrates have been decimated in the past and are nowadays confined to highly fragmented and human-dominated landscapes [1]–[4]. Consequently, managers and ecologists face common “metapopulation” scenarios in which it is necessary to counteract the detrimental effects of genetic, demographic and environmental stochasticity [5]. However, even when unoccupied patches of appropriate habitat exist and past causes of extirpation have been identified and corrected, the paucity of extant populations often limits connectivity among small and isolated fragments, thus preventing rescue effects and natural (re)colonisations. In these situations, management strategies often have to rely on reintroduction programmes to create viable metapopulations [4].

Metapopulation restoration is highly dependent on dispersal behaviour [6], and this trait is known to be affected by a variety of environmental, social and individual parameters [7]. Although little is known for long-lived vertebrates, studies in other animals have shown that dispersal patterns may differ between (re)introduced or translocated individuals and their native counterparts 8–10. This poses additional difficulties in anticipating transfer rates among local populations and estimating the effect of reintroductions both on release patches and the entire metapopulation. Since juvenile (natal) dispersal is generally more important than adult (breeding) dispersal [11], and emigrations from the (re)introduction site usually occur soon after release [10], movements of individuals early in life may be especially informative. Moreover, pre-breeding dispersal in large vertebrates with deferred maturity usually takes several years during which individuals wander over extensive areas which, in turn, affect survival prospects [12], . Consequently, the estimation of the magnitude of these pre-settlement movements and the comparison of patterns between introduced and wild individuals are a key element for the design of an adaptive management of programs aiming to restore viable metapopulations.

During the last two centuries, the bearded vulture (*Gypaetus barbatus*) was extirpated from most of the European mountain ranges such that at the end of the twentieth century the species remained confined mainly to the Pyrenees, and to a lesser extent to the Balkans, Corsica and Crete where all together less than 120 territories were occupied [14], [15]. The concern for the species has been translated into heavy economical outlays from regional, national and European budgets. Only Life-nature programs have invested more than 95 million euros between 1994 and 2008 [16]. Within this context, from 1986 onwards bearded vultures have been successfully reintroduced in the Alps, were 20 territories where active in 2010 [17], [18]. Another reintroduction began in 1996 (with first releases in 2006) in southern Spain where breeding has not yet occurred [19]. In the short and medium term, other reintroduction projects in several European mountains and in Mediterranean islands have been proposed to minimize metapopulation extinction risks. This is probably the only way to increase the distribution range of the species in Europe because the largest natural European population is confined to a sole mountain range (Pyrenees) and because almost none geographical expansion of this population has occurred during the last decades [20].

The bearded vulture can therefore be considered a model of transnational efforts to rebuild a viable metapopulation, in this case of a long-lived vertebrate confined to mountain patches. As it is well-established, the future viability of demes inhabiting single patches and the whole metapopulation will be clearly dependent on the flow of dispersing individuals [21]. Nonetheless, the inherent difficulty in long-term monitoring of long-lived species has largely precluded the collection of reliable information on these subjects [22]. Here, by contrasting existing information on the pre-breeding dispersal movements of GPS tracked wild and reintroduced bearded vultures inhabiting the mountain ranges of Central and Western Europe, we offer a first insight into the potential connectivity among populations. We hypothesize that, apart of individual differences inherent to age and sex [23], asymmetries could also appear between wild and reintroduced populations. Particularly, we predict shorter movements in the Pyrenean (wild) birds as consequence of different types of factors such as conspecific attraction and modification of habitat quality through supplementary feeding [21], [24].

## Methods

### Ethics Statement

All the work has been conducted in accordance with relevant national and international guidelines, and conforms to the legal requirements. Birds reintroduced in Andalusia and the Alps born within an international captive-breeding program (European Endangered Species) have been marked before the release. In the case of wild individuals (Pyrenean population), captures and blood sample collection have been carried out in compliance with the Ethical Principles in Animal Research. Thus, protocols, amendments and other resources have been done according to the guidelines approved by each Autonomous government following the R.D.1201/2005 (10^th^ October 2005, BOE 21^st^ October 2005) of the Ministry of Presidency of Spain.

### Study Area and General Methods

**Figure 1:**
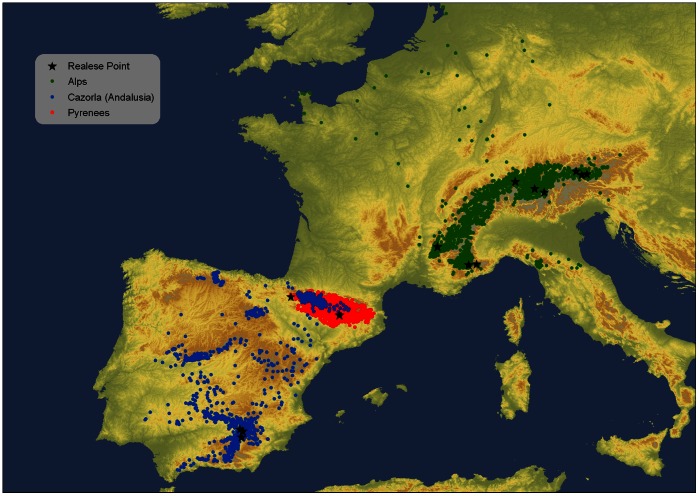
Distribution of mean-day locations of individual satellite-tracked pre-adult bearded vultures of the wild Pyrenean population (*n* = 9), and the reintroduced populations of Andalusia (*n* = 13) and the Alps (*n* = 24). Stars show release points.

Patterns of movements of non-breeding bearded vultures were obtained from Platform terminal transmitters (PTT) and GPS-GSM satellite tags tagged to: i) wild born non-breeding individuals from the Spanish Pyrenees (*n = *9), and ii) captive-bred, reintroduced birds to the Andalusian mountains (Spain, *n* = 13) and Alps (France, Italy, Austria and Switzerland, *n* = 24). Pyrenean birds were captured by means of radio-controlled bow-nets at feeding stations (*n* = 7) or as fledglings in the nests (*n* = 2). All the immature birds were aged (calendar year) on the basis of plumage characteristics. Birds reintroduced in Andalusia and the Alps were born within an international captive-breeding program (European Endangered Species). All the individuals were placed at release points when they were around 90–100 days old but fledging did not take place until birds were around 120 days old. In Andalusia three close release points were placed in the Cazorla Mountains whereas in the Alps, birds were released at six points distributed in France (two points, nine birds), Italy (two points, two birds), Austria (three points, five birds) and Switzerland (two points, eight birds) (Fig. 1). All birds from Spain and one bird from the Alps were fitted with solar-powered 70gr Argos/GPS PTTs (Microwave Telemetry Inc, Maryland, USA). The remaining birds from the Alps were fitted either with PTT-100 105 gr LC4 PTTs (two birds) or with battery (13 birds) and solar-powered (eight birds) GPS-GSM satellite tags (GPS PLUS Bird, VECTRONIC Aerospace GmbH, Berlin, Germany).

Transmitters from the Pyrenees were fixed to the birds as backpacks with a Teflon harness with a central ventral rupture point. Birds from the Alps and the Pyrenees were fixed with a Teflon leg harness with a dorsal rupture point [25]. Blood samples were taken from the brachial vein to determine the birds’ sex using standard PCR-based molecular techniques [26].

Vultures were monitored from 2006 to 2012 in the Pyrenees (mean = 744.56 days; range = 95–1769 days), Andalusia (mean = 765.69 days; range = 236–2268 days) and Alps (mean = 383.96 days; range = 193–756 days, see Table S1). For the two reintroduced populations and in the case of the two young marked in the Pyrenees in their nests, we excluded data obtained prior to September 1 to focus only on periods in which birds had already completed their flying skills [15]. To evaluate vultures’ movements, we selected the mean GPS locations per bird per day. There are a number of parameters adequate to quantify movements associated with dispersal behaviour [27]. Within them, we considered two variables evaluating respectively “search rate” and “philopatry of search”, namely: i) step length, estimated as the distance travelled per day by each individual between consecutive days; and ii) total dispersal distance, estimated as the distance between mean daily locations and the point of origin (i.e., releasing or trapping points for reintroduced and wild birds, respectively).

We look for differences in vulture movements among subpopulations (Pyrenees, Andalusia, Alps) using Generalized Linear Mixed Models (logarithm link function and gamma error distribution). We included individual age (covariate) and sex (fixed factor) in the models to account for potential inter-individual variability. Because data suggest a hump-shaped pattern, candidate models were fitted by considering age both as a linear and a quadratic term. Because we had repeated measures for the same birds over different years, we included individual identity nested within subpopulation as well as year as random terms. Individual effects were modelled with two different covariance structures: variance component and first-order autoregressive. This variance components covariance structure is the best fitting according to −2 Log likelihood (results not shown) so all models were built accordingly. Models were performed using SAS 9.2 [28] with a Laplace approximation and a between-within method for computing the denominator degrees of freedom.

## Results

**Figure 2:**
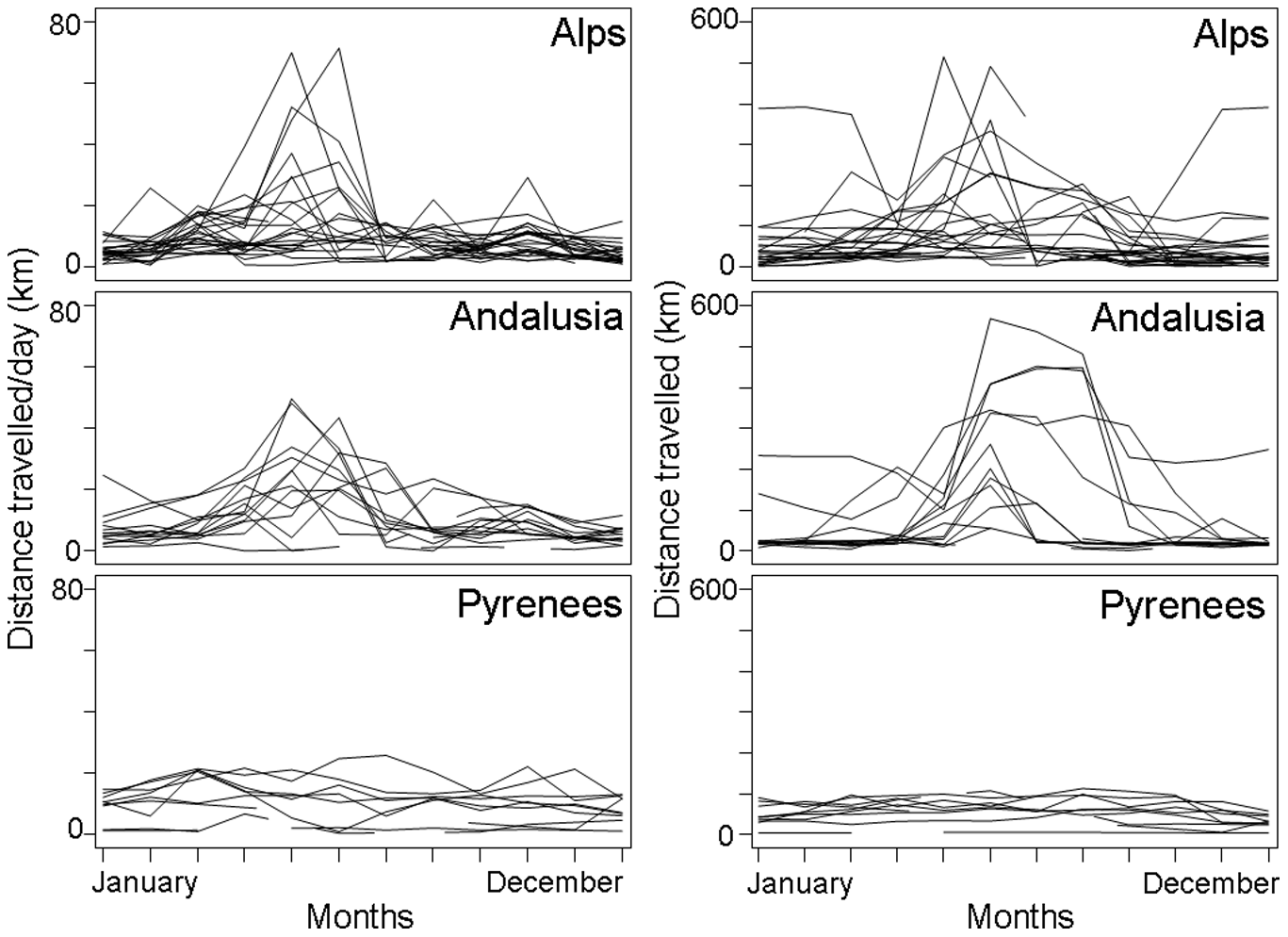
Movements of pre-adult bearded vultures through the year-cycle in the wild Pyrenean population and the reintroduced populations of Andalusia and the Alps. Each line represents an individual.

All the movements of the nine wild bearded vultures marked in the Pyrenees were restricted to these mountains (Fig. 1). Maximum distances from the natal (*n* = 2) or capture point (*n* = 7) ranged from 7.49 to 196.95 km (mean = 52.65, s.d. = 22.80, *n* = 9). On the contrary, birds released in Andalusia and Alps performed frequent long-distance travels (maximum distance for Andalusian birds: range = 24.22–609.60 km, mean = 84.23, s.d. = 76.30, *n* = 13; maximum distance for Alpine birds: range = 48.70–869.08 km, mean = 67.73, s.d. = 46.67, *n = *24). All the Iberian range was visited by the Andalusian birds, including the Cantabrian Mountains and the Pyrenees. Bearded vultures released in the Alps moved through most of these mountains, especially the Western regions, rarely visiting neighbouring mountains ranges as the northern Apenines and the Massif Central (Fig. 1). It is worth noting that other individuals left the Alps for short periods and flew to the European plain and the North Sea coast. Data on vulture’s movements were obtained after monitoring birds during long time periods that in most cases (30 out of 46 birds) covered a whole year cycle and, thus, potential seasonal variations (mean tracking time for birds ∼ 1.54 years; Fig. 2).

**Figure 3:**
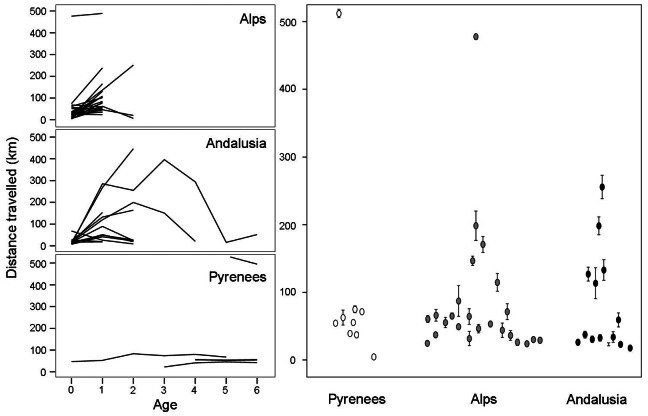
Interpopulation variability in daily average distances travelled by pre-adult bearded vultures. Left: daily average distances travelled according to age classes. Right: average (± s.e.) differences among subpopulations.

Models show that movement patterns differed between wild birds from the Pyrenees and birds reintroduced into the other two regions but also between different age classes. Step length showed a quadratic relationship with age (estimate age = 0.57, s.e. = 0.10; F_1,38_ = 0.06, p = 0.81; estimate for age^2^ = −0.07, s.e. = 0.01, F_1,38_ = 33.39, p<0.0001; Fig. 3), without differences between males and females (F_1,26_ = 0.33, p = 0.57). Raw data show that distances between successive days (i.e., step length) appeared to be of a similar range among birds from the three subpopulations (Alpine birds: 3.91–19.18 km; Andalusian birds: 1.85–19.18 km; Pyrenean birds: 1.95–20.01 km). However, individuals from the three subpopulations showed significant differences in step lengths (F_2,45_ = 8.09, p = 0.0010), birds of Alps and Andalusia being similar (LSMEANS statement: t = −1.01, d.f. = 45, p = 0.31) but different from Pyrenean birds (LSMEANS: Pyrenees and Alps : t = 3.48, d.f. = 45, p = 0.0011; Pyrenees and Andalusia: t = 4.01, d.f. = 45, p = 0.0002).

**Figure 4:**
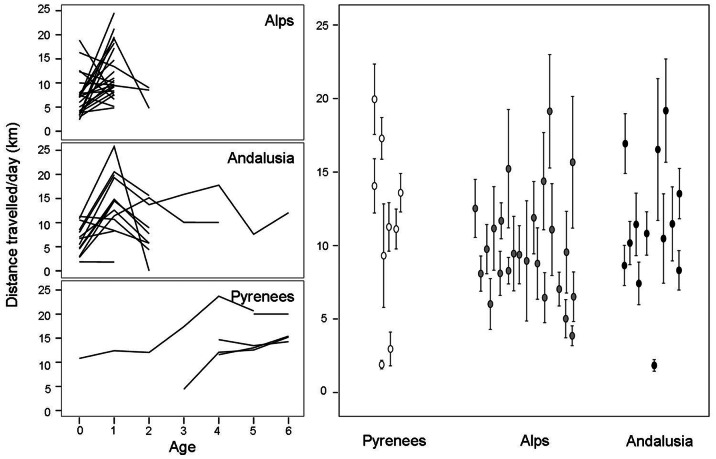
Interpopulation variability in the distances covered by pre-adult bearded vultures with respect to the capture or release point (capture for the Pyrenees and release for the individuals from the Alps and Andalusia). Left: average distances covered according to age classes. Right: average (± s.e.) differences among subpopulation.

Total dispersal distance also followed a quadratic relationship with age (estimate age = 0.93, s.e. = 0.90; F_1,38_ = 1.07, p = 0.31; estimate for age^2^ = −0.16, s.e. = 0.01, F_1,38_ = 201.76, p<0.0001; Fig. 4), without sex-related differences (F_1,26_ = 0.03, p = 0.87). Differences were also significant between individuals from the different subpopulations (F_2,45_ = 9.66, p = 0.0003), birds from Alps and Andalusia showing similar dispersal distances (LSMEANS: t = −1.15, d.f. = 45, p = 0.26) but differing from those of the Pyrenees (Pyrenees and Alps : t = 3.78, d.f. = 45, p = 0.0005; Pyrenees and Andalusia: t = 4.35, d.f. = 45, p<0.0001).

## Discussion

Our results clearly show significant differences in step length and total dispersal distance between bearded vultures belonging to the Pyrenees and the Alps and Andalusia mountains, without significant differences between the last two. Thus, we can support our hypothesis that birds of the wild and reintroduced populations differed in their movement patterns, with shorter dispersal among the wild ones. This pattern remains significant even when accounting for the significant effect of age on dispersal distances.

In the past, bearded vultures occupied most of the European mountainous ecosystems, including low ranges near sea level. Thus, we could expect that pre-adult bearded vultures would not be restricted in their movements because of a lack of suitable habitat when released in southern Europe. In fact, in the 19th century Spanish bearded vultures were largely distributed in medium and small mountains of the Iberian Peninsula [14]. However, according to our predictions, our results confirm that wild pre-adult bearded vultures from the Pyrenean population moved over much smaller areas than those birds reintroduced into Andalusia and the Alps. This can have important conservation consequences because it affects potential flow of individuals among the most important Palearctic subpopulations, thus compromising large-scale conservation strategies.

Our findings could be a consequence of different, non-mutually exclusive factors. First, it could be argued that the heterogeneity in age-class distributions among subpopulations is responsible for differences in animal movements. In fact, most wild birds from the Pyrenees were older than those of reintroduced populations. Although further studies including individuals’ long-term monitoring should be performed to properly separate age effects on movement patterns, information based on long-term monitoring programs carried out in Pyrenees with marked individuals of all ages (*n* = 106) show that these vultures have never been located outside this mountain range [29]. Another factor causing differences observed in our study could be bird origin (i.e., wild vs captive-bred). Captivity can relax selective pressures, change the direction of selection or impose completely novel pressures either intentionally or inadvertently provoking noticeable behavioural changes [30]. An experimental approach, where movement patterns of wild and reintroduced birds are compared in the same population, would confirmed whether bird origin is responsible for these differences in movement patterns or not. However, as it is not feasible to perform this approach with this long-lived endangered species in a near future, we would just suggest this hypothesis as a potential explanation. Finally, another not mutually exclusive explanation for differences in movement patterns among populations would be related to the reduction in the population size and range of the species. The precipitous decline of the European bearded vulture population may have had strong genetic consequences whose role in the observed differences should be taken into account. Previous studies show genetic differences between bearded vultures from the Pyrenees and those of the captive pool [31]–[33]. A genetic drift altering the genetic composition of the remnant Pyrenean population after the demographic bottleneck of the last century has also been registered [33]. As other previous studies supported a genetic basis for dispersal [34], it could be that genetic differences found in the Pyrenean population are linked to individual dispersal propensity, as we discuss below. In addition, human activities can alter selective environments disassociating certain behavioural or life-history decisions and outcomes normally coupled with them, creating evolutionary traps so that rapid environmental changes result in maladaptive behavioural decisions [35]. In our case, long-term human persecution may have selected for individuals that do not disperse from natal areas, which might have put them at lower risk for being shot or poisoned in remote high-risk areas.

Not independent from this last point and in agreement with our predictions, human manipulation of the environmental carrying capacity may also play an important role in the observed movement patterns. Availability of food resources seems to be high in all the study areas [17], [19], [36], [37] so that food shortages would not explain the observed differences in movement patterns. However, it could be at least partially related to long-term supplementary feeding programs carried out in the Pyrenean region [38]. Regional administrations have created a huge network of supplementary feeding points in the central Pyrenees where dozens of birds concentrate [29]. As a result, new recruits settle near existing pairs to the extent that average neighbouring distance has decreased by about 20% in the last ten years [20], [39]. In addition to this philopatric behaviour, demographic pressure leads some adult male birds to sneak into existing territories, forming polyandrous trios [40]. In turn, the resulting high density reached by the bearded vulture population in the Central Pyrenees [39] may be reinforcing conspecific attraction [29], [41]. Ultimately, the concatenation of these factors would contribute to the inability of the Pyrenean bearded vulture population to expand as seen during the last decades [20].

Reintroduction programs aim to establish a species in an area that was once part of its historical range but from which it has become extinct, using wild-caught or captive-bred individuals [42], [43]. The results and goals of these programs however are still hotly debated because in many cases their convenience, justification, and usefulness remain unclear [44]. In particular, the origin of reintroduced birds (captive vs wild) is controversial. Captive programs may be designed to attenuate potentially low genetic diversity [45] but may have negative side effects, because introduced non-local alleles may cause a population to become less suited to local environmental conditions by producing intermediate phenotypes [46], [47]. On the other hand, wild-reared individuals may also be affected by genetic constraints when the variability of source populations is low [48]. Our results show marked differences in dispersal ability between wild and reintroduced birds and open discussion on how to manage the whole European metapopulation of this emblematic species in the future. In particular it will be necessary to determine if the low dispersal ability of the Pyrenean population is a local adaptation or the result of contemporary population decimation and habitat manipulation promoted within conservation programs. Differences in step length and total dispersal distance were significant among wild and reintroduced populations, although more marked for the second dispersal estimator. This suggests that all the individuals have the potential to move long distances, but whereas wild birds moved within a restricted area reintroduced vultures wandered over larger areas including not only release zones but also other mountain ranges of the western Mediterranean basin and even the great European plain. Whatever the reason, it seems clear that in a current scenario of decreasing persecution and high availability of resources, the creation of other populations of individuals with a high dispersal propensity can help to geographically expand the species beyond its current localized distribution range.

## Supporting Information

Table S1 **Bearded vultures** ***Gypaetus barbatus*** **tracked in the different study areas, including information about the age of the individuals when they were captured or released, their sex (determined by molecular techniques), the period during which movements were surveyed, the total number of days tracked and total number of locations.**
(DOC)
