# Supplementary material for: Uneven Large-Scale Movement Patterns in Wild and Reintroduced Pre-Adult Bearded Vultures: Conservation Implications
Source: PLoS One. 2013 Jun 11;8(6):e65857. doi: 10.1371/journal.pone.0065857 (PMC3679195; doi:10.1371/journal.pone.0065857)
Supplement: Table S1 — Bearded vultures Gypaetus barbatus tracked in the different study areas, including information about the age of the individuals when they were captured or released, their sex (determined by molecular techniques), the period during which movements were surveyed, the total number of days tracked and total number of locations. (DOC) [file pone.0065857.s001.doc]

**Table S1**

| **Individual** | **Age** | **Sex** | **Area** | **Tracking period (day-month-year)** | **Days** | **N Locations** |
| --- | --- | --- | --- | --- | --- | --- |
| PYR1 | 4 | Male | Pyrenees | 14-05-09/30-11-11 | 578 | 3780 |
| PYR2 | 5 | Female | Pyrenees | 06-05-08/27-02-10 | 467 | 5512 |
| PYR3 | 6 | Male | Pyrenees | 25-11-10/28-02-11 | 71 | 3344 |
| PYR4 | 5 | Male | Pyrenees | 23-11-10/24-02-12 | 318 | 2063 |
| PYR5 | 3 | Female | Pyrenees | 14-05-09/10-09-12 | 814 | 4339 |
| PYR6 | 0 | Male | Pyrenees | 08-11-07/11-09-12 | 1257 | 6132 |
| PYR7 | 6 | Male | Pyrenees | 03-06-09/25-02-12 | 156 | 2891 |
| PYR8 | 0 | Male | Pyrenees | 02-09-07/05-06-08 | 117 | 575 |
| PYR9 | 4 | Female | Pyrenees | 06-05-08/28-02-11 | 640 | 4606 |
| ALP1 | 0 | Male | Alps | 01-10-07/05-07-09 | 329 | 701 |
| ALP2 | 0 | Female | Alps | 02-09-09/26-06-10 | 201 | 422 |
| ALP3 | 0 | Male | Alps | 02-09-10/27-09-11 | 373 | 550 |
| ALP4 | 0 | Female | Alps | 02-09-09/12-09-10 | 251 | 492 |
| ALP5 | 0 | Female | Alps | 10-09-07/30-05-08 | 180 | 288 |
| ALP6 | 0 | Female | Alps | 01-09-08/08-11-09 | 303 | 634 |
| ALP7 | 0 | Male | Alps | 01-09-10/26-09-12 | 701 | 8232 |
| ALP8 | 0 | Male | Alps | 01-09-11/26-09-12 | 283 | 2034 |
| ALP9 | 0 | Female | Alps | 01-09-10/21-09-12 | 686 | 1332 |
| ALP10 | 0 | Female | Alps | 01-09-10/02-06-11 | 250 | 376 |
| ALP11 | 0 | Male | Alps | 01-09-11/26-09-12 | 358 | 3411 |
| ALP12 | 0 | Female | Alps | 02-09-09/29-08-10 | 240 | 476 |
| ALP13 | 0 | Female | Alps | 01-09-11/07-09-12 | 306 | 2425 |
| ALP14 | 0 | Male | Alps | 01-09-07/31-08-08 | 232 | 451 |
| ALP15 | 0 | Male | Alps | 01-09-07/21-07-08 | 194 | 345 |
| ALP16 | 0 | Male | Alps | 01-09-10/08-05-12 | 500 | 656 |
| ALP17 | 0 | Female | Alps | 01-09-11/26-09-12 | 257 | 1220 |
| ALP18 | 0 | Male | Alps | 01-09-11/24-09-12 | 314 | 2420 |
| ALP19 | 0 | Male | Alps | 01-09-10/05-04-11 | 216 | 356 |
| ALP20 | 0 | Female | Alps | 01-09-11/10-06-12 | 195 | 1761 |
| ALP21 | 0 | Female | Alps | 01-09-10/13-03-11 | 181 | 287 |
| ALP22 | 0 | Male | Alps | 01-09-11/29-03-12 | 136 | 857 |
| ALP23 | 0 | Female | Alps | 02-09-09/27-06-10 | 207 | 416 |
| ALP24 | 0 | Female | Alps | 01-09-06/02-05-07 | 94 | 701 |
| AND1 | 0 | Female | Andalusia | 01-09-10/16-11-12 | 719 | 3112 |
| AND2 | 0 | Female | Andalusia | 01-09-09/16-11-12 | 478 | 2641 |
| AND3 | 0 | Male | Andalusia | 01-09-06/17-05-08 | 471 | 2017 |
| AND4 | 0 | Male | Andalusia | 01-09-06/1705-08 | 762 | 3585 |
| AND5 | 0 | Male | Andalusia | 01-09-10/18-11-12 | 756 | 3641 |
| AND6 | 0 | Male | Andalusia | 03-09-06/16-07-07 | 195 | 1973 |
| AND7 | 0 | Female | Andalusia | 01-09-09/07-05-10 | 229 | 1088 |
| AND8 | 0 | Female | Andalusia | 01-09-09/07-05-10 | 329 | 2039 |
| AND9 | 0 | Male | Andalusia | 02-09-07/06-05-11 | 752 | 2131 |
| AND10 | 0 | Female | Andalusia | 01-09-10/24-04-08 | 704 | 3057 |
| AND11 | 0 | Female | Andalusia | 01-09-07/24-04-08 | 116 | 608 |
| AND12 | 0 | Male | Andalusia | 01-09-06/16-11-12 | 1335 | 5244 |
| AND13 | 0 | Male | Andalusia | 01-09-10/22-07-12 | 334 | 1702 |
